# Embarking on a new journey, exploring infinite possibilities—Welcoming the 2025 edition of *Animal Models and Experimental Medicine*

**DOI:** 10.1002/ame2.12571

**Published:** 2025-02-05

**Authors:** Chuan Qin

**Affiliations:** ^1^ Chinese Association for Laboratory Animal Sciences Beijing China; ^2^ Institute of Laboratory Animal Sciences Chinese Academy of Medical Sciences & Peking Union Medical College Beijing China

Against the backdrop of a new era that commands the attention of scholars worldwide, and on a scientific research journey filled with both hope and challenges, *Animal Models and Experimental Medicine* (*AMEM*), as a beacon guiding innovation and rigorous research in the field of animal models and experimental medicine, has once again shone brightly in 2024. With an impact factor of 3.8, a JCI and JCR Q2 ranking, and a CiteScore of 6.3, the journal has demonstrated a continuous rise in its influence and academic status.

In 2024, the number of submissions doubled to over 400, covering 59 countries and regions, with full‐text views exceeding 350 000, spanning 216 countries and regions. Numerous manuscripts were cited by top international journals. To promote discipline development and exchange, we expanded content to 190 pages and organized six special issues covering cutting‐edge fields such as stem cells, cardiovascular diseases, and bone regeneration. We not only provided a broad stage for groundbreaking research but also built an efficient, cutting‐edge, and welfare‐ethical academic exchange platform for scholars worldwide, facilitating international academic communication and dissemination of frontier knowledge.

Furthermore, we successfully convened an editorial board meeting, bringing together experts and scholars from 18 countries and regions to delve into issues such as journal development plans and optimization of peer review processes, aiming to better serve the scientific community. Based on this, we decided to change the publication frequency of the journal to monthly starting in 2025, providing scholars with more timely and comprehensive academic information through a more frequent publication cycle.

Notably, in response to critical medical health issues, we will launch a special issue on oncology in 2025, aiming to gather the latest research findings in the field of oncology from both domestic and international perspectives, providing robust scientific support for cancer prevention and treatment. Meanwhile, we will strengthen the study, promotion, and implementation of standards in the field of laboratory animals, enhancing the scientific and normative use of laboratory animals to safeguard the healthy development of experimental medicine.

Here, we also sincerely solicit ideas and contributions from practitioners in the field of laboratory animal science and technology and experimental medicine from around the world, covering articles on scientific research management, welfare ethics, and laboratory animal resources. We look forward to scholars sharing valuable experiences and resources to promote resource sharing and utilization, jointly advancing the progress and development of laboratory animal science and technology. Additionally, we will establish a special column to introduce outstanding research teams and leading enterprises, aiming to deepen industry exchanges. Professionals are welcome to actively submit manuscripts and contribute to the prosperity of the discipline.

Dear readers, authors, and fellow researchers, standing at a new starting point, we are fully aware that the road ahead is filled with both opportunities and challenges. However, it is these challenges that inspire us to keep moving forward. Let us move forward hand in hand with greater enthusiasm and firmer conviction to jointly create an even more glorious future for *AMEM*!

Lastly, we sincerely wish everyone good health, smooth work, and fruitful research achievements in the new year!

*AMEM* Editor‐in‐Chief 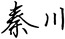


